# Suppressor of cytokine signaling 3 (SOCS3) is not an independent biomarker of colorectal adenoma risk

**DOI:** 10.1186/1756-0500-3-144

**Published:** 2010-05-25

**Authors:** Kathryn E Hamilton, P Kay Lund, Joseph A Galanko, Robert S Sandler, Temitope O Keku

**Affiliations:** 1 Department of Cell and Molecular Physiology, University of North Carolina School of Medicine, 111 Mason Farm Road, Chapel Hill, North Carolina 27599-7545, USA; 2 Department of Medicine and Center for Gastrointestinal Biology and Disease, University of North Carolina School of Medicine, 130 Mason Farm Road, Chapel Hill, North Carolina 27599-7545, USA

## Abstract

**Background:**

Inflammation and its associated pathologies are increasingly suggested as risk factors for colorectal cancer (CRC) development. Previous research from our group has shown that increased levels of circulating, pro-inflammatory cytokines IL-6 and TNFα promote colorectal adenoma risk. Emerging data in mice and humans suggest that Suppressor of Cytokine Signaling 3 (SOCS3) may act as a tumor suppressor in the intestine, and decreased SOCS3 expression may promote CRC. As SOCS3 has been shown to inhibit the actions of IL-6 and TNFα in the intestine, we hypothesized that decreased SOCS3 expression in normal mucosa may predispose to adenomas and thus increase risk for CRC.

**Findings:**

We examined SOCS3 mRNA levels in normal mucosa biopsies of 322 screening colonoscopy patients (93 with adenoma and 229 without adenoma) using real-time qRT-PCR. Logistic regression analysis was used to generate odds ratios (OR) and 95% confidence intervals to determine if low SOCS3 expression was associated with adenoma status. Median SOCS3 values did not differ between patients with or without adenoma. Logistic regression analysis showed no association (unadjusted or adjusted for age and sex) between SOCS3 and colorectal adenomas.

**Conclusions:**

Low SOCS3 mRNA expression is not an independent biomarker of colorectal adenoma risk in the normal mucosa. SOCS3 silencing likely occurs later in CRC progression.

## Introduction

Recent evidence in mice and humans suggest that the anti-inflammatory protein Suppressor of Cytokine Signaling 3 (SOCS3) may act as a tumor suppressor in the colon [1,2]. Specific silencing of SOCS3 expression in intestinal epithelial cells (IEC) increased tumor load in the azoxymethane/dextran sodium sulfate (AOM/DSS) mouse model of inflammation-associated CRC [1]. Furthermore, SOCS3 expression is low or silenced by promoter hyper-methylation in other cancers, including lung, liver, and squamous cell carcinoma [3-5].

Prior studies from our group demonstrated that increased systemic levels of pro-inflammatory cytokines IL-6 and TNFα correlate with risk of colorectal adenoma [6]. SOCS3 has been shown to limit the actions of both of these cytokines as well as their downstream targets STAT3 and NFκB, which are frequently activated in humans and mouse models of CRC [2,7-12]. Based on these studies, we investigated SOCS3 mRNA expression in the normal mucosa of patients undergoing routine screening colonoscopy to determine if low SOCS3 predisposes to adenoma and could thus be considered an early biomarker of CRC risk.

## Materials and methods

### Study Population and Data Collection

All eligible subjects provided written, informed consent. Consenting participants were enrolled in Diet and Health Study IV at University of North Carolina Hospitals as previously described [13]. Briefly, subjects undergoing routine colonoscopy provided rectal biopsies and blood samples for the study. Subjects also consented to follow-up interview to collect diet and lifestyle information. High quality RNA from 322 subjects (93 with adenoma, 229 without adenoma) with complete information on plasma IL-6 and TNFα, age, race, sex, waist hip ratio (WHR), family history and non-steroidal anti-inflammatory drug (NSAID) use was assayed for SOCS3. Patients in the adenoma group were defined as having one or more adenomas by the study pathologist based on standard criteria. The study was approved by the University of North Carolina School of Medicine Institutional Review Board.

### RNA Extraction and Real-Time qRT-PCR

RNA from four pooled, normal colon biopsies per subject was extracted using Qiagen's RNeasy kit (Valencia, CA) and reverse transcribed with AMV-Reverse Transcriptase (Promega) as previously described [13].

SOCS3 mRNA abundance was determined using the ABI Prism 7900 HT (Applied Biosystems, Foster City, CA) and Platinum Quantitative PCR SuperMix-UDG (Invitrogen, Carlsbad, CA). Human SOCS3 (NM_003955.3) was quantified using Applied Biosystem primer/probe set targeting exon 2. The housekeeping gene hydroxymethylbilane synthase (HMBS, NM_000190.3) was chosen as a low-abundance, invariant control. Standard curves for each primer/probe set were generated using gel-isolated, sequence-confirmed PCR products. Cycling included initial denaturation at 95°C for 5 minutes followed by 45 cycles of 95°C denaturation for 15 seconds and 60°C annealing for 45 seconds. Threshold cycles analysis was performed using Applied Biosystem SDS v2.2.2 software and values are expressed as copy number relative to HMBS. All PCR runs included standards and inter-run calibrator controls (pooled sample cDNA), as well as non-reverse transcribed (no-RT) and water controls. Samples were run in triplicate.

### Statistical Analysis

Means and standard errors were generated for continuous variables, and frequencies and percentages were generated for categorical variables. T-tests and Mann-Whitney tests were used to compare cases with controls on continuous variables. SOCS3 values were log-transformed to normalize the distribution. Chi-Square tests were used to compare cases and controls on categorical variables. Logistic regression was used to test for an association between case/control status and SOCS3. Levels of SOCS3 were categorized into tertiles based on control values. Age, race, sex, WHR, NSAIDS, IL-6, TNFα, and family history were assessed as potential confounders of SOCS3-adenoma association. Each variable was put into a model separately with SOCS3, and if one of the two dummy variables for SOCS3 changed by at least 15% compared to when only SOCS3 was in the model, then that co-variable passed the first stage for being a confounder. All such variables were then entered into a model with SOCS3 and a backwards, stepwise regression was done with the SOCS3 variable being forced into the model. Only age and sex met these criteria for confounding and thus they were included in the final model.

## Results

Descriptive characteristics of the population in the SOCS3-adenoma study are shown in Table 1. Consistent with results from our prior reports for the study population, subjects with adenomas were older, more likely to be male, had higher waist-hip ratios and increased plasma IL-6 [6,13]. There was no difference in median SOCS3 between individuals with or without adenoma.

**Table 1:** Descriptive Characteristics of Study Participants

Variable	Case (n = 93)	Control (n = 229)	p-value *
Age median (1^st^, 3^rd ^quartile)**	58 (51, 56)	55 (50, 63)	0.03
Race-White N (%)†	73 (78)	177 (77)	0.88
Sex-Male N (%)	51 (55)	85 (37)	0.004
WHR median (1^st^, 3^rd ^quartile)	0.922 (0.854, 1.000)	0.888 (0.804, 979)	0.04
Family History of CRC - N (%)	16 (20)	31 (15)	0.37
Plasma IL-6 median (1^st^, 3^rd ^quartile)	0.21 (0.00, 0.56)	0.07 (0.00, 0.41)	0.01
Plasma TNFα median (1^st^, 3^rd ^quartile)	1.93 (1.44, 2.74)	1.75 (1.30, 2.44)	0.14
Plasma CRP median (1^st^, 3^rd ^quartile)	3,959 (1,934, 14,278)	7,239 (2,294, 16,728)	0.14
Tissue SOCS3 median (1^st^, 3^rd ^quartile)	3.09 (2.33, 3.77)	3.09 (2.35, 3.80)	0.77

* P-values are from Fishers Exact Test for categorical variables and Mann-Whitney test to compare medians for continuous variables.

** Standard error (se); Waist Hip Ratio (WHR); Suppressor of Cytokine Signaling 3 (SOCS3)

† Percentages within columns

To determine if low SOCS3 is associated with having an adenoma, odds ratios and 95% confidence intervals were generated using logistic regression analysis (Table 2). There was no difference in odds ratios in subjects in the lower tertiles of SOCS3 values, indicating that low levels of local SOCS3 expression do not associate with adenoma risk.

**Table 2:** Adjusted Association Between Local SOCS3 Levels and Adenoma

Tissue SOCS3	Case/Control*	**OR (95% CI)**†
Tertile 3 (36.4-180.7)‡	28/69	1.0 (Reference)
Tertile 2 (13.8-36.4)	27/72	0.9 (0.5, 1.7)
Tertile 1 (0.6-13.8)	29/69	1.0 (0.5, 1.8)

*Adjusted for age and sex

†Odds ratio (OR) and 95% confidence interval (CI); odds of having colorectal adenomas

‡Tertile cut-offs based on distribution of SOCS3 among control subjects; tertile 3 was used as reference

## Discussion

Identifying local factors that predispose patients to early, pre-cancerous lesions may make it possible to stratify screening based on risk, but risk factors that occur early in CRC development are not well defined. Our group has shown that patients with adenoma have reduced apoptosis in their normal mucosa, demonstrating a field effect that predisposes the individual to a higher risk of developing precancerous adenomas [14,15]. We hypothesized that SOCS3 might be a good biomarker of colorectal neoplasia risk, but contrary to expectation the study was negative.

Patients with inflammatory bowel diseases (Crohn's disease or ulcerative colitis) have an increased risk of developing CRC, which is associated with the degree and duration of intestinal inflammation (reviewed in [16]). In addition, recent data suggest that individuals with chronic, low levels of inflammation (such as increased circulating IL-6 and TNFα) have increased odds of having adenoma and thus increased CRC risk [6]. SOCS3 is an anti-inflammatory protein that limits IL-6 induction of STAT3, as well as TNFα induction of NFκB. IEC-specific silencing of SOCS3 leads to a dramatic increase in tumor load in a mouse model of inflammation-associated CRC [1]. Furthermore, recent studies show that STAT3 activation is increased, and SOCS3 is silenced in tumors of patients with ulcerative colitis-associated and sporadic CRC [2,17]. The current study tested the hypothesis that patients with adenoma may have lower SOCS3 in the normal mucosa than patients without adenoma, thus contributing to a permissive environment for aberrant growth. However, our study found that low or silenced SOCS3 expression does not occur in the normal mucosa of patients with colorectal adenoma.

One potential limitation of the study is possible effect of standard bowel preparation on SOCS3 expression. While we have not directly tested this for SOCS3, similar studies of other genes have shown that there is no significant change in mucosal gene expression in patients using bisacodyl or polyethylene glycol bowel preparations [18]. Another possible limitation is that SOCS3 mRNA levels do not reflect changes in SOCS3 phosphorylation, which leads to its targeting for proteasomal degradation [19]. However, other studies show that SOCS3 is silenced by DNA hyper-methylation in CRC tumors [2], indicating that evaluating changes in gene expression is an appropriate measure of SOCS3 in this study. Finally, while our results could indicate that SOCS3 is more important in the underlying pathogenesis of inflammation-associated rather than sporadic CRC, recent studies comparing SOCS3 expression in both ulcerative colitis-associated and sporadic tumors found that SOCS3 was decreased and there was no significant difference between the two groups for SOCS3 [2]. Taken together with these studies, we conclude that SOCS3 silencing occurs later in the progression from adenoma to adenocarcinoma, and is not an independent, early biomarker of CRC risk in the normal mucosa.

## Competing interests

The authors declare that they have no competing interests.

## Authors' contributions

KEH carried out the molecular studies, participated in data analyses, and drafted the manuscript. PKL and RSS participated in the study conception, design, and coordination. JAG performed the statistical analyses. TOK participated in the study conception, design, and coordination, and helped to draft the manuscript. All authors read and approved the final manuscript.
